# Neuromodulatory Systems and Their Interactions: A Review of Models, Theories, and Experiments

**DOI:** 10.3389/fncir.2017.00108

**Published:** 2017-12-22

**Authors:** Michael C. Avery, Jeffrey L. Krichmar

**Affiliations:** ^1^SNL-R, Systems Neurobiology Laboratory, Salk Institute for Biological Studies, La Jolla, CA, United States; ^2^Department of Cognitive Sciences, University of California, Irvine, Irvine, CA, United States; ^3^Department of Computer Science, University of California, Irvine, Irvine, CA, United States

**Keywords:** neuromodulation, computational neuroscience, computational modeling, brain disorders, neuromodulatory systems

## Abstract

Neuromodulatory systems, including the noradrenergic, serotonergic, dopaminergic, and cholinergic systems, track environmental signals, such as risks, rewards, novelty, effort, and social cooperation. These systems provide a foundation for cognitive function in higher organisms; attention, emotion, goal-directed behavior, and decision-making derive from the interaction between the neuromodulatory systems and brain areas, such as the amygdala, frontal cortex, hippocampus, and sensory cortices. Given their strong influence on behavior and cognition, these systems also play a key role in disease states and are the primary target of many current treatment strategies. The fact that these systems interact with each other either directly or indirectly, however, makes it difficult to understand how a failure in one or more systems can lead to a particular symptom or pathology. In this review, we explore experimental evidence, as well as focus on computational and theoretical models of neuromodulation. Better understanding of neuromodulatory systems may lead to the development of novel treatment strategies for a number of brain disorders.

## Introduction

**Figure 1:**
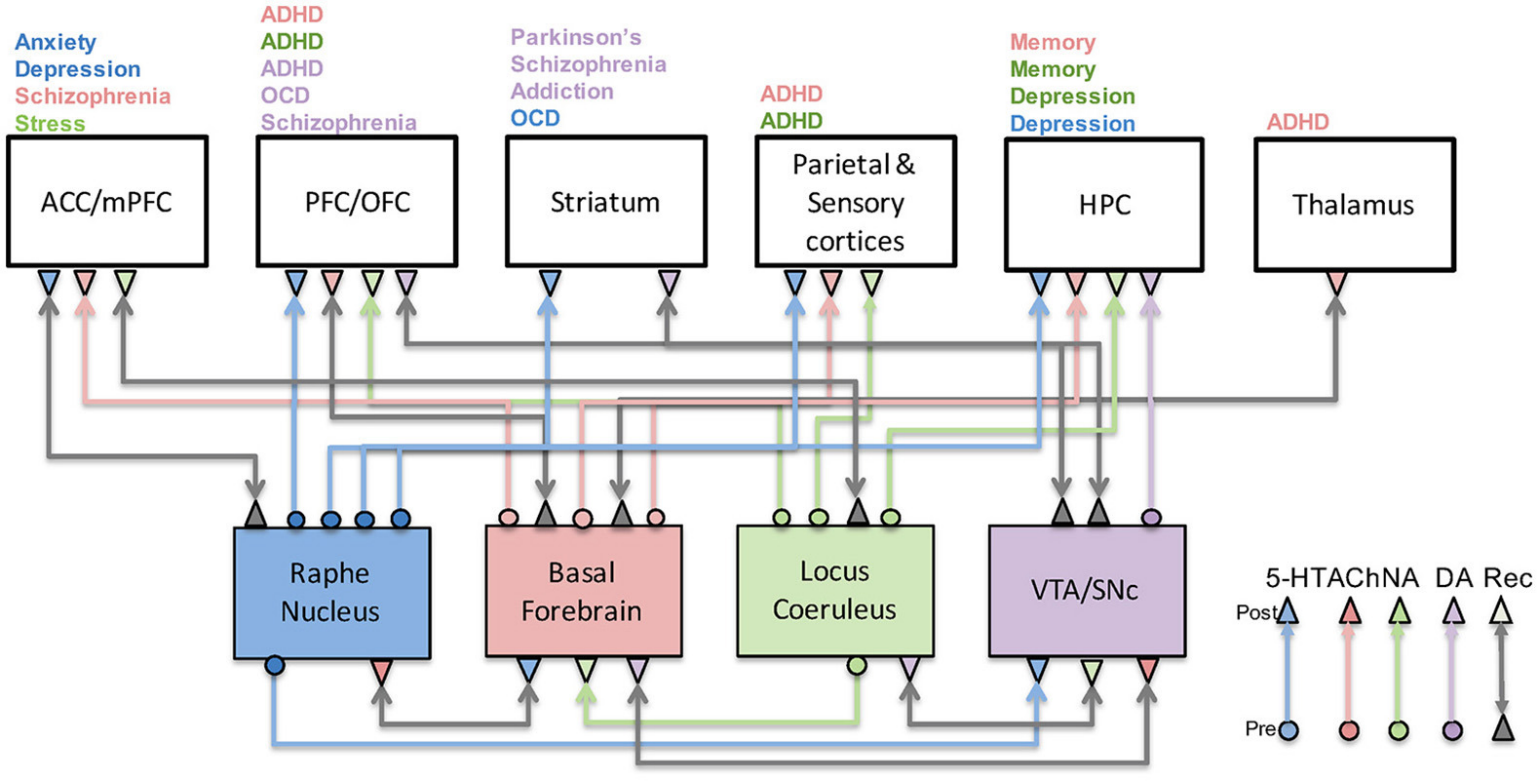
Neuromodulatory system interactions and their role in disease. This figure illustrates how serotonergic (blue), cholinergic (red), noradrenergic (green), and dopaminergic (purple) systems are highly connected to one another as well as cortical and subcortical structures. Their malfunction has been associated with a host of neurological and psychiatric conditions as indicated above each brain region. Gray arrows denote recurrent connections.

The mammalian neuromodulatory system consists of small pools of neurons (on the order of thousands in the rodent and tens of thousands in the human) located in the brainstem, pontine nucleus, and basal forebrain, which can have a powerful effect on cognitive behavior. Ascending neuromodulatory systems include noradrenergic, serotonergic, dopaminergic, and cholinergic projections from the brainstem and basal forebrain regions to broad areas of the central nervous system (Briand et al., 2007). Neuromodulators signal risks, rewards, novelty, effort, and social cooperation. These systems provide a basis for many higher cognitive functions; attention, decision-making, emotion, and goal-directed behavior result from the interaction between the neuromodulatory systems and brain areas, such as the anterior cingulate, frontal cortex, hippocampus, sensory cortex, and striatum (Figure 1). In this review, we explore experimental evidence, with a strong focus on computational and theoretical models of neuromodulation. We discuss how these models might increase our understanding of brain disorders.

## Dopaminergic system

The dopaminergic neuromodulatory system has been extensively studied and is involved in nearly every aspect of brain function from cognition to behavior (Schultz, 1997; Schultz et al., 1997, 2000; Berridge, 2004, 2012; Hyman et al., 2006; Durstewitz and Seamans, 2008). Dopamine originates in either the ventral tegmental area (VTA) or substantia nigra pars compacta (SNc). A substantial amount of research has gone into understanding the circuits that regulate dopamine neuron firing as well as the downstream effects of dopamine release. In particular, we know that the VTA and SNc are strongly influenced by the striatum and subcortical structures such as the lateral habenula and pedunculopontine tegmental nucleus. It has been shown that the phasic increase and dip in dopamine response are due to the activation of the pedunculopontine tegmental nucleus and lateral habenula, respectively (Matsumoto and Hikosaka, 2007; Hong and Hikosaka, 2014). Phasic increases also may be due to collicular or other sensory or non-sensory inputs to VTA/SNc when a salient event is identified (Redgrave and Gurney, 2006).

Direct and indirect pathways in the striatum disinhibit and inhibit dopamine neuron firing, respectively, and are themselves modulated by cortical and limbic inputs. Prefrontal and hippocampal inputs to the striatum disinhibit the VTA leading to an increase in phasic and tonic activity, respectively (Takahashi et al., 2011; Murty et al., 2017). It has been hypothesized that an abnormal increase in glutamatergic input to striatum leads to excess dopamine in the striatum and may account for symptoms of schizophrenia (de la Fuente-Sandoval et al., 2011). Computational models of the basal ganglia have also shed light on the role dopamine plays in Parkinson's disease (Moustafa and Gluck, 2011; Moustafa et al., 2013; Balasubramani et al., 2015). Still, many questions remain regarding cortical and limbic inputs to the striatum, how they compete to drive striatum responses, and how phasic and tonic dopamine levels might regulate these brain regions. Understanding these upstream effects is a critical component as we develop a circuit-level understanding of brain disorders that are thought to result from abnormal dopaminergic activity.

Dopamine neurons, in turn, send projections to the striatum, thalamus, amygdala, hippocampus, and prefrontal cortex, demonstrating the “feedback” nature of this circuit. Dopaminergic neurons originating in the SNc project to the dorsal striatum. Abnormalities in this pathway can lead to motor disorders including Parkinson's disease. Two distinct areas in the VTA project to either the ventral striatum (mesolimbic) or to the prefrontal cortex (mesocortical). The effect that dopamine has on its downstream target depends on the post-synaptic receptor and the firing mode of the DA neuron. Phasic release of dopamine in the striatum, for example, preferentially activates D1 receptors on striatal Medium Spiny Neurons (MSNs) and increases their activity (direct pathway). Increases in tonic dopamine, on the other hand, are thought to activate D2 receptors (D2R) in the striatum, which inhibit MSNs in the striatum (indirect pathway). It has recently been shown, however, that phasic DA can also lead to increases in inhibitory post-synaptic currents in D2R-MSN neurons (Marcott et al., 2014), suggesting the role of tonic and phasic dopamine may be more complex than originally thought. Increases in both phasic and tonic activity would, therefore, lead to an increase in the direct pathway and a decrease in the indirect pathway, which would ultimately cause a strong release of inhibition on the thalamus.

The effects of tonic and phasic dopamine in the prefrontal cortex appears to be opposite of the striatum. D1 receptors in the prefrontal cortex are preferentially activated by tonic dopamine and have an inverted-U dose-dependent response on superficial neurons (discussed below), whereas D2 receptors are activated by phasic dopamine and increase the activity of subcortically-projecting neurons in deep layers. This suggests that D2 receptors play a preferential role in behavior and reward processing, whereas D1-expressing neurons are involved in working memory and attentional modulation of visual cortices (Noudoost and Moore, 2011; Gee et al., 2012; Puig and Miller, 2014). Interestingly, the temporal dynamics of the phasic responses of dopaminergic cells resemble those found in a machine learning method known as reinforcement learning (Schultz et al., 1997). As we discuss below, this gives us a more rigorous understanding of the function of dopaminergic neurons in the brain and helps to understand the important components of dopamine responses for normal and abnormal brain function.

## Models of dopaminergic function

**Figure 2:**
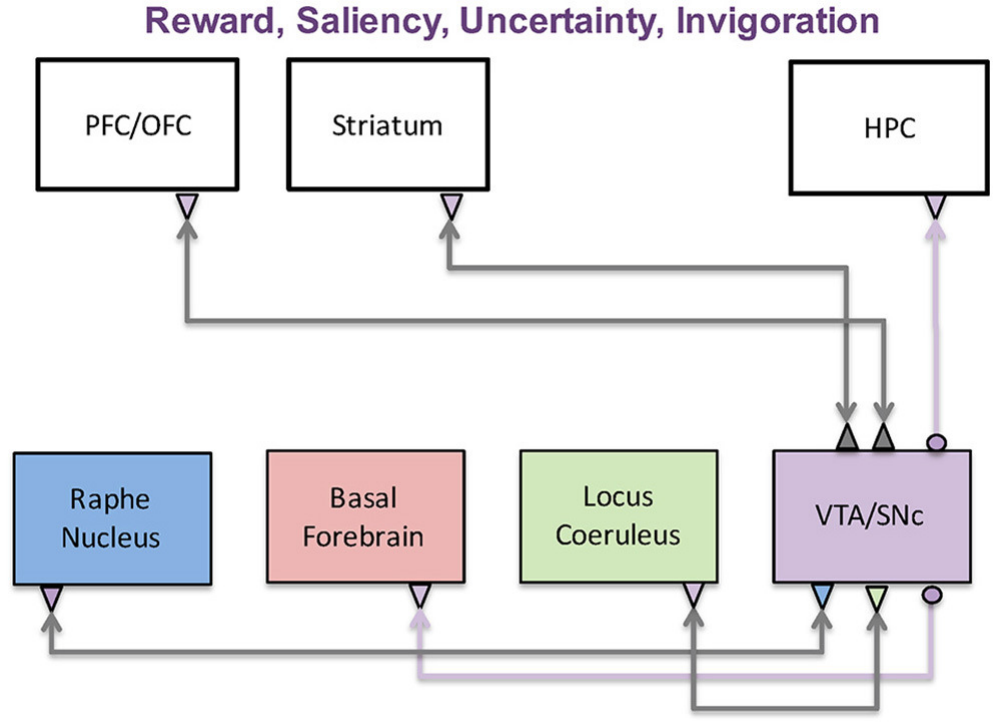
The dopaminergic system and its functions. The dopaminergic system, which originates in the VTA and SNc, has been implicated in a wide variety of functions including reward, saliency, uncertainty, and invigoration. These functions are achieved through interactions with the prefrontal cortex, striatum, and hippocampus. It is also reciprocally connected with the three other neuromodulatory systems, further complicating its role in disease states.

The responses of dopaminergic neurons during behavioral conditioning experiments closely resemble temporal difference reward prediction error variables found in reinforcement learning. This has led to the prediction error hypothesis of dopamine signaling, which connects dopamine signaling to reinforcement learning models and indicates that dopamine neurons play an important role in human decision-making. It has also been hypothesized that dopaminergic neurons respond to and broadcast uncertainty and/or novelty related signals. The circuits involved in these computations are shown in Figure 2. We discuss these circuits and theoretical models below, together with several computational, network-based models that propose mechanisms for how dopamine-related computations are implemented in the brain.

## Reinforcement learning models

Reinforcement learning is a machine learning method that concerns itself with finding the appropriate actions that maximize future reward. Formally, the theory aims to find an optimal function, or policy, (*P*) for mapping states (*S*) into actions (*A*) that maximize the sum of future reward. Temporal difference learning methods, such as the actor-critic model, solve this problem by computing a reward prediction error signal (δ), which is used in the updating of a value function (reward expectation) and policy as shown in the equations below.

(1) $$\begin{array}{l} \delta_{t}=r_{t+l}+\gamma V\left(s_{t-1}\right)-V\left(s_{t}\right)\\ \;V(s_{t+1})=V\left(s_{t}\right)+\alpha \cdot \delta \\ \;P(a|s_{t+1})=P(a|s_{t})+\alpha \cdot \delta \end{array}$$

where *r*_*t*+__1_ is the observed reward at time *t*+1, *V(S*_*t*_*)* is the value of state *S* at time *t*, γ is a discounting factor, and α is the learning rate. The algorithm works by sampling the environment, making predictions, and then adjusting the predictions based on the error signal. The ability to use the prediction error signal to update value estimates and behavioral policies is what gives this algorithm (and organisms) the flexibility to adapt to a dynamic environment. The temporal dynamics of the δ term closely resembles responses seen in dopaminergic cells *in vivo*, suggesting a prediction error hypothesis of dopamine function (Schultz, 1997; Schultz et al., 1997).

Doya extended the temporal difference equations to other neuromodulatory systems (Doya, 2002, 2008). In his view, dopamine signals the error in reward prediction (δ in Equation 1), serotonin controls the discounting of reward prediction (γ in Equation 1), and acetylcholine controls the speed of memory update (α in Equation 1). More recent theoretical models have extended the temporal difference rule to other neuromodulatory systems and have attributed the α parameter, which controls the rate of learning, to the serotonergic (Balasubramani et al., 2015) or noradrenergic systems (Nassar et al., 2012).

Abnormalities in dopaminergic responses have been linked to a host of disorders, including schizophrenia, attention and mood disorders, and Parkinson's disease (Wise, 2004; Björklund and Dunnett, 2007; Schultz, 2007; Sillitoe and Vogel, 2008). Within the context of the reinforcement-learning framework, these disorders are thought to arise from a failure of dopaminergic cells to properly compute reward prediction errors and communicate them to downstream structures. For example, depressive symptoms would result from a reduction in reward sensitivity within the reinforcement-learning framework (Huys et al., 2013; Chen C. et al., 2015). Abnormalities in reward prediction errors could also induce positive symptoms of schizophrenia (delusions/hallucinations) through the construction of unusual associations and abnormal internal models of the world (Maia and Frank, 2011). As discussed below, different hypotheses of dopamine function can lead different conclusions regarding the manifestation of a particular disease.

## Dopamine, uncertainty, and novelty

Although theoretical and experimental evidence suggests that dopamine neurons encode reward prediction error (Schultz et al., 1997), several lines of evidence suggest that this hypothesis is incomplete. First, dopaminergic neurons not only respond to reward and reward prediction, but also respond to any salient or novel input in the environment regardless of its reward value (Bromberg-Martin et al., 2010). Second, the response of dopamine neurons to reward predicting stimuli is too fast to be mediated by a “predictive” input that would likely originate in prefrontal cortices (Redgrave and Gurney, 2006). Third, dopamine depletion primarily impacts task performance and learning is left intact (Berridge and Robinson, 1998; Cannon and Palmiter, 2003; Berridge, 2012).

This has led to several alternative hypotheses regarding dopaminergic function. The two we describe below are the saliency and uncertainty hypotheses. The saliency hypothesis suggests that dopamine neurons respond to salient or novel environmental events to discover novel actions (Redgrave and Gurney, 2006). This directly contrasts with the prediction error hypothesis in which reward prediction errors were used to update the weights of a set of defined actions. Within this framework, abnormal dopaminergic responses would lead to abnormalities in processing salient information. This is consistent with the aberrant salience hypothesis of schizophrenia (Kapur, 2003), which suggests that positive symptoms in schizophrenia originate and evolve from an improper allocation of attentional resources to what normally would be non-salient events.

It has also been suggested that dopamine encodes the precision, the inverse of uncertainty, of alternative actions beliefs (Friston et al., 2012). This hypothesis is rooted in Bayesian inference models and is able to reconcile the prediction error hypothesis and incentive salience hypothesis, which accounts for the fact that dopamine is not necessary for learning. If dopamine encodes precision values, abnormal dopamine responses would lead to false inferences about the world as a result of an improper balance of sensory and prior information. False inferences could ultimately manifest as positive symptoms of schizophrenia, including delusions and hallucinations (Adams et al., 2013).

The uncertainty and salience hypotheses predict that dopamine plays an important role in regulating the information that gains access to conscious perception. The mechanism by which this is achieved, however, is unknown. Previous theoretical and computational models, as well as experimental studies have suggested several mechanisms that could support such computations, including: dopaminergic projections to the prefrontal cortex/basal ganglia, balance of excitation/inhibition in prefrontal cortex, D1/D2 receptor activation, and NMDA/GABA receptor activation. In particular, Cohen and colleagues developed a model that suggests that dopamine acts as a gate to regulate information that can enter prefrontal cortex (Braver and Cohen, 1999). In this model, dopamine acts on both the afferent excitatory and local inhibitory input in the prefrontal cortex, leading to a disruption in the maintenance and updating of information in the prefrontal cortex. They suggested that cognitive symptoms in schizophrenia arise from increasing the variability of dopamine inputs to the prefrontal cortex, which would destabilize working memory traces (Braver and Cohen, 1999; Rolls et al., 2008). This model was extended to include the basal ganglia as part of the gating mechanism (Hazy et al., 2006).

**Figure 3:**
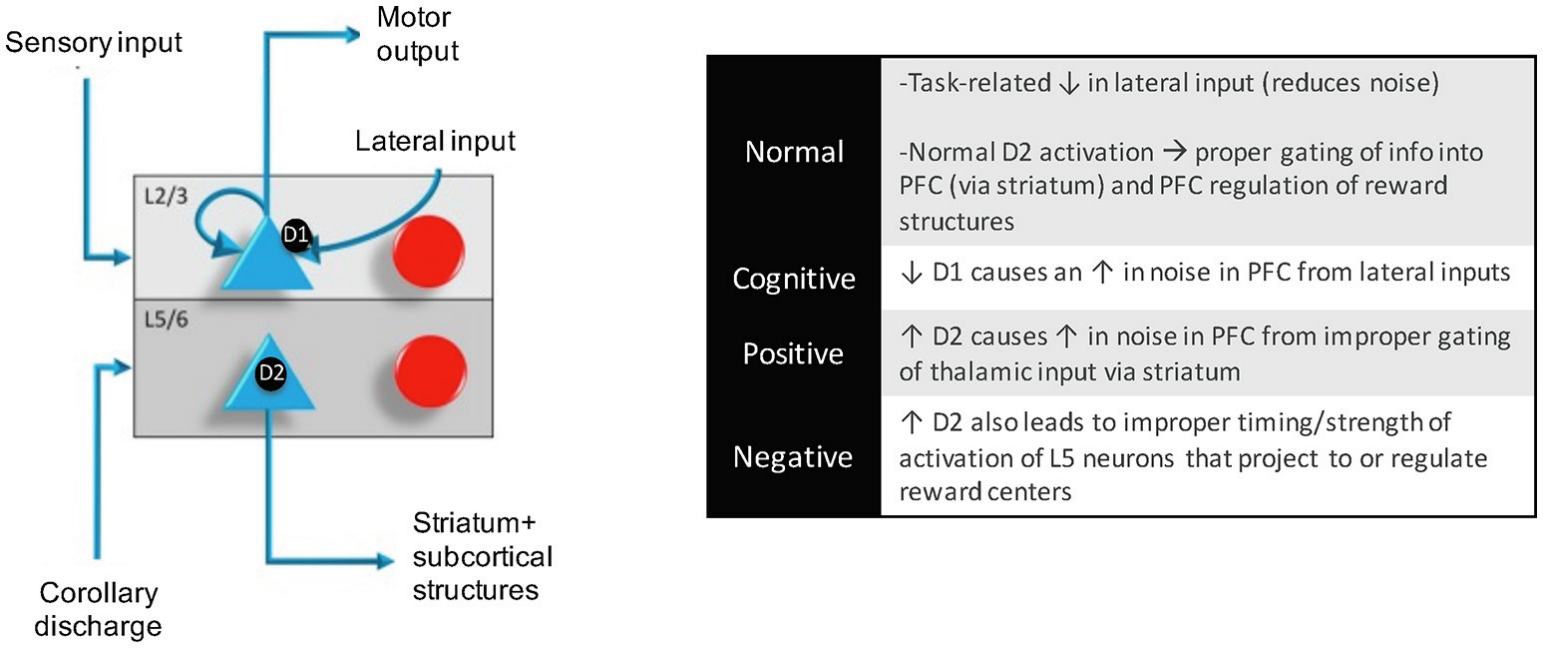
Within a column in the PFC, neuromodulators were modeled by changing the strength of inputs from non-preferred directions (D1 receptors) between layer 2/3 neurons in different columns and the strength layer 5 neuronal responses (D2 receptors). This architecture also shows how layer 5 neurons in each column received input from the MD/SC and output to a non-specific inhibitory group and the basal ganglia in order to clear working memory and update other columns, respectively. In this model, Positive and negative symptoms arise from abnormalities in layer 5 outputs to subcortical structures and cognitive symptoms arise from a “leaky” spread of excitation of lateral excitatory inputs within the PFC.

More recently, we developed a circuit-based model that shows how D1 and D2 receptors could balance the relative weight of information from different brain regions (Avery and Krichmar, 2015). This computational model suggests that activation of D1 receptors allows information from the thalamus to take precedence within the prefrontal cortex by blocking interference from lateral excitation in superficial layers (see Figure 3). Optimal D1 activation results in one column of the PFC being active, which represents holding a stimulus in working memory. Low D1 activation results in inter-columnar interference within the PFC. This can lead to a noisy representation of an object in working memory via lateral input from other regions of the PFC, which might manifest as cognitive symptoms in schizophrenia. A similar mechanism is proposed for attention disorders (Arnsten et al., 2012). Our model also suggests that activation of D2 receptors on deep layers 5 neurons in the prefrontal cortex disinhibits thalamic inputs to the prefrontal cortex via interactions with the basal ganglia (Avery and Krichmar, 2015). Improper activation of D2 receptors in the prefrontal cortex may lead to non-specific activity from the thalamus, potentially contributing to positive symptoms observed in schizophrenics. We also suggest that improper activation of D2 receptors on subcortically projecting layer 5 neurons leads to abnormalities in reward processing, resulting in negative symptoms of schizophrenia.

A model based on a dynamical systems framework suggests that D1 and D2 receptors influence the stability of persistent and spontaneous cortical attractor states by increasing and decreasing NMDA and GABA conductances, respectively (Durstewitz and Seamans, 2008). If a network is in a stable regime (high D1, low D2), the pattern of neuronal activity in the network will remain unchanged until a sufficiently strong input can push the network into a different state. If the network is in an unstable regime (low D1, high D2), however, even weak inputs impinging on the network will cause neurons to randomly shift from spontaneous to persistent states. This is related to the gating hypothesis in the sense that a highly stable state would effectively block incoming information (closed gate), whereas an unstable state would allow inputs to drive the network into a different state (open gate). The dynamical systems model predicts that instabilities in cortical attractor states, which arise from an improper balance in D1 and D2 receptor activation, might lead schizophrenia symptoms (Loh et al., 2007; Durstewitz and Seamans, 2008). In particular, cognitive and negative symptoms result from reduced NMDA (reduced D1), which leads to a reduction in firing rate in the prefrontal cortex. A reduction in both NMDA and GABA, on the other hand, leads to instabilities in the network that produce positive symptoms.

Each of these computational models offers insight into understanding the role of the dopaminergic system in the healthy and diseased brain and alludes to possible treatment strategies. The dynamical systems model, for example, suggests that NMDA and GABA receptors are important for maintaining stable working memory representations and may be important targets for drug therapies. Network-based models, on the other hand, point to regions of interest for deep brain stimulation or pharmacological intervention and could also make predictions regarding downstream effects of manipulation of a particular region of the brain. These models will become even more important as we begin to develop experiments that connect different levels of investigation of the brain and will allow us to generate more refined hypotheses regarding disease mechanism and treatment strategies.

## Serotonergic system

**Figure 4:**
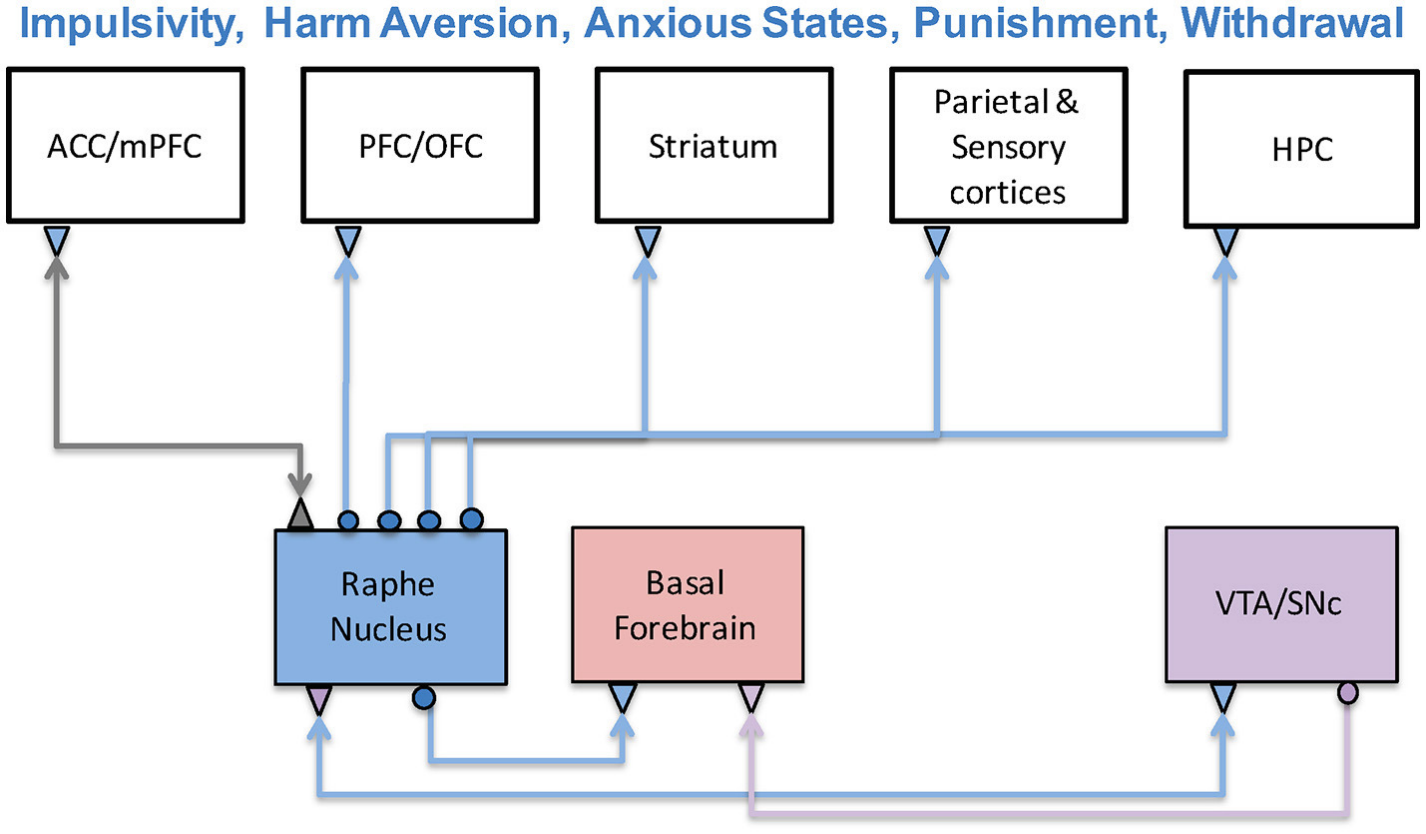
The serotonergic system and its functions. The serotonergic system originates in the Raphe Nucleus of the brainstem and is connected to prefrontal, sensory, limbic, and striatal structures. Serotonin has been associated with a variety a functions including impulsivity, harm aversion, anxious states, punishment, and withdrawal. Experimental and theoretical studies have suggested it has an antagonistic relationship with the dopaminergic system.

Serotonergic projections, which originate in the raphe nuclei of the brainstem, extend to almost all forebrain areas (Barnes and Sharp, 1999), including the cortex, ventral striatum, hippocampus, and amygdala (Harvey, 2003; Meneses and Perez-Garcia, 2007). The raphe receives strong connections from the prefrontal cortex and the anterior cingulate cortex (Briand et al., 2007). Through interactions with these brain regions and other neuromodulatory systems, serotonin influences a broad range of decision-based functions such as reward assessment, cost assessment, impulsivity, harm aversion, and anxious states (Asher et al., 2013). The circuits involved in these functions are shown in Figure 4. Impairments to the serotonergic system have been linked to anxiety disorders and depression (Craske and Stein, 2016), as well as Parkinson's disease (Bédard et al., 2011).

## Serotonin and impulsivity

Several studies have investigated serotonin's involvement in impulsivity, which is the tradeoff between taking an immediate reward, or else waiting for a future, potentially larger reward. In the temporal difference learning rule, this term is called temporal discounting or gamma (see γ in Equation 1). Kenji Doya suggested that serotonin levels may be related to temporal discounting level (Doya, 2002). His group has confirmed this prediction in rodent and human experiments (Tanaka et al., 2007; Miyazaki et al., 2011). In addition, it has been shown that forebrain serotonin depletion the steepens discounting of delayed rewards, which leads to impulsive actions (Winstanley et al., 2003). In another study, it was observed that higher serotonin firing activity causes a rat to wait longer for upcoming rewards, as predicted by temporal discounting (Miyazaki et al., 2011). Wait errors associated with lower serotonergic neural activity suggest that 5-HT can affect choice involving delayed rewards.

The link between serotonin and temporal discounting has been explored using the Acute Tryptophan Depletion (ATD) procedure. 5-HT requires the amino acid tryptophan, which only can be acquired through diet. In ATD, subjects temporarily have a low-protein diet and drink an amino acid supplement that omits tryptophan. In essence, ATD acts as a temporary serotonin lesion. Altering 5-HT levels via ATD influences a subject's ability to resist a small immediate reward over a larger delayed reward (Tanaka et al., 2007, 2009; Schweighofer et al., 2008). As such, subjects that underwent ATD had both an attenuated assessment of delayed reward and a bias toward small reward, which were indicative of impulsive behavior and higher temporal discounting.

## Serotonin and harm aversion

Serotonin (5-HT) has been linked to predicting punishment or harm aversion (Cools et al., 2008; Crockett et al., 2008, 2012; Seymour et al., 2012). ATD caused subjects to be aggressive and risk taking by rejecting more monetary offers in the Ultimatum Game (Crockett et al., 2008). In a reversal-learning task, Cools and colleagues demonstrated that ATD subjects made more errors for harmful than rewarding stimuli (Cools et al., 2008). Crockett and colleagues showed that lowering 5-HT levels with ATD resulted in decreased punishment-induced inhibition in a Go/No-Go task to Crockett et al. (2009). In a follow up ATD study, they investigated the mechanisms through which 5-HT regulated punishment-induced inhibition with their Reinforced Categorization task (Crockett et al., 2012). Furthermore, recent evidence suggests that enhancing serotonin function through serotonin specific reuptake inhibitors (SSRIs) increased harm aversion, while enhancing dopamine through levodopa reduced altruism (Crockett et al., 2015). Together, these results suggest that 5-HT influences the ability to inhibit actions that predict punishment and to avoid harmful circumstances.

## Serotonin and anxiety

In addition to punishment and impulsivity, 5-HT affects stress and anxiety (Millan, 2003; Jasinska et al., 2012). It has been proposed that environmental impact factors and genetic variations of the serotonin transporter (5-HTTLPR) can be linked to stress (Jasinska et al., 2012). Furthermore, 5-HT function has been tied to an organism's anxious states triggered by conditioned or unconditioned fear (Millan, 2003). This suggests a functional role for 5-HT in the control of anxious states. These anxious states and behavioral responses were modeled in neurorobot experiments, which will be described in more detail in the Dopamine and Serotonin Opponency section. In brief, a stressor caused the robot's simulated serotonin level to increase, which in turn caused the robot to hide (Krichmar, 2013). In the model, artificially decreasing the rate that serotonin returned to base levels had a similar effect to the short allele variant of 5-HTTLPR discussed above, where serotonin reuptake is impaired. Under these conditions, the neurorobot showed longer-lasting hiding responses to a stressful sensor event (e.g., a bright light). These responses are similar to those seen in mice, where manipulations of 5-HT1A and 5-HT2A receptors resulted in the mice avoiding the center of an open arena and exploring novel objects, suggesting that these manipulations of serotonin led to higher anxiety levels (Heisler et al., 1998; Weisstaub et al., 2006).

## Models of serotonin neuromodulation

**Figure 5:**
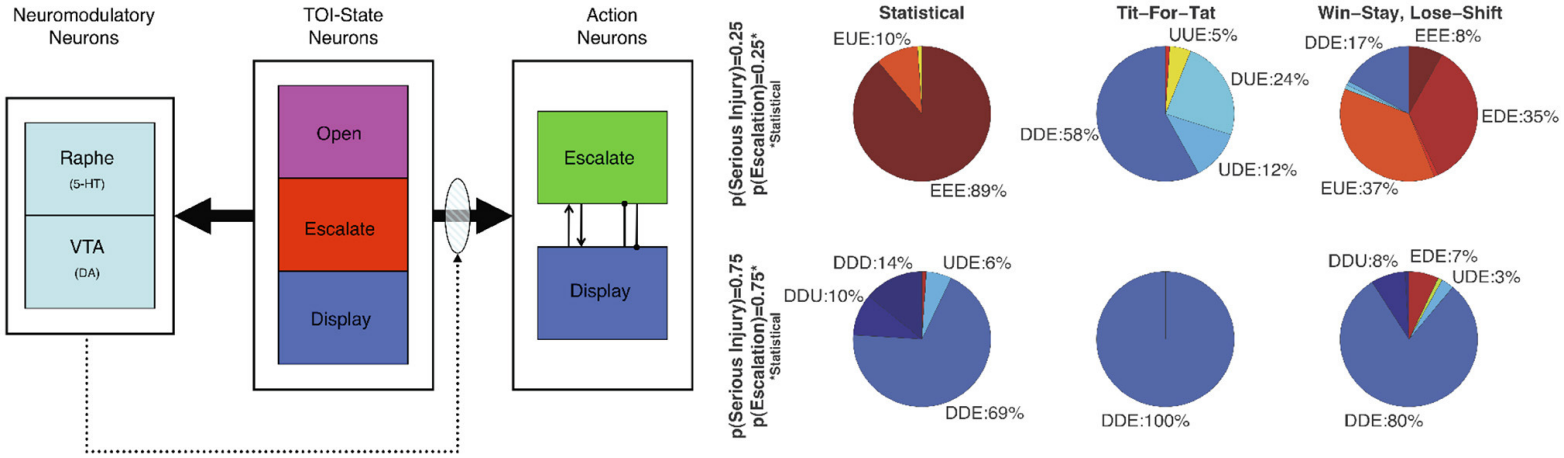
Neuromodulation effects in simulation of the Hawk-Dove game. **(Left)** Architecture of the neural model (Neuromodulatory: Raphe and VTA; TOI-State: Open, Escalate, and Display; and two Action: Escalate and Display). Solid arrows from the TOI-State neurons denote all-to-all connections. The shaded oval and dotted arrows denote plastic connections. Within the Action neurons, the arrowhead denotes an excitatory connection, and the line with the dot at the end denotes an inhibitory connection. **(Right)** Proportion of actions taken by the Neural agent. Open, Escalate, and Display are states the Neural agent observes, and Escalate (E), Display (D), and Undecided (U) are actions the Neural agent can take. U represents random choice between “E” and “D”. Labels denote the Neural agent's response to the three states. Dove-like strategies are displayed in blue, Hawk-like are displayed in red, and the lack of a strong bias is displayed in yellow. Reproduced from Asher et al. (2010) with permission.

Using an Actor-Critic model, Asher et al. (2010), Zaldivar et al. (2010) constructed a neural network where a reward critic represented the dopaminergic system and a cost critic represented the serotonergic system (see Figure 5). In these experiments, the neural network model played the socioeconomic game of Hawk-Dove against other agents. In the Hawk-Dove game, players must choose to either take a resource (escalate) or share a resource (display). If both players escalate, a fight ensues, resulting in a penalty. If only one player chooses to escalate, then that player gets the resource, and the other player get nothing. If both players display, then the resource is shared. The reward critic tracked the expected value of obtaining the resource, and the cost critic tracked the expected punishment from fighting for the resource.

The simulations showed that the model was sensitive to the other player's strategy and the game environment (i.e., the likelihood of receiving a serious injury). The adaptive neural agent was more likely to escalate over the resource when activity of the reward system (VTA) exceeded the activity of the cost system (Raphe). Conversely, when the reward activity did not exceed the activity of cost, the adaptive neural agent tended toward display actions. The simulations also predicted that impairment of the serotonergic system would lead to perseverant, uncooperative behavior. A simulated lesion of the serotonergic system resulted in the agent almost always engaging in risk taking (or lack of harm aversion) behavior, which was similar to behavior seen in human studies where serotonin levels were lowered via ATD while subjects played games such as Prisoner's Dilemma and the Ultimatum game (Wood et al., 2006; Crockett et al., 2008).

Following the simulation studies, human robot interaction experiments were performed to test the model's performance against human players, as well as the influence of embodied agents on game play (Asher et al., 2012). These experiments involved ATD; the dietary manipulation described above that temporarily lowers serotonin levels. Overall, subjects demonstrated aggressive behavior when playing against an aggressive version of the model with a simulated 5-HT lesion, which tended to escalate more. This resulted in subjects altering their strategy from Win-Stay-Lose-Shift (WSLS) against agents, to a retaliatory Tit-For-Tat (T4T) against an aggressive version of the model. A Bayesian analysis revealed two types of subjects; one in which subjects were more aggressive when tryptophan-depleted, and one in which they were less aggressive. In addition, some of the subjects were more aggressive toward robots than simulations, and vice versa (Asher et al., 2012). These results highlight the importance of taking individual variation into consideration in serotonin studies.

In a model inspired by serotonergic neuromodulation related to punishment or harm, Weng and colleagues constructed a neural model where artificial serotonin levels regulated stress or pain in two different tasks (Weng et al., 2013). The first was a visual recognition task that investigated how such a system can learn visual cues via a teacher that only provides punishments and reward signals. The second task had an agent wander in the presence of a friend and a foe. In both tasks, the interplay between reward and pain led to high performance and the emergence of internal representations without the need of a supervisory signal.

These computational models show how simulating serotonergic effects, even in fairly simple neural models, explain how altering serotonin modulation of neural activity can affect harm aversion and altruistic behavior. Moreover, embodying these models in robots highlights these behaviors and leads to the possibility of using human robot interaction as a means to study these disorders.

At the neuronal level, detailed computational models that include ionic currents can investigate receptor specific effects of serotonin to drug treatments (Wong-Lin et al., 2012; Cano-Colino et al., 2014). In a model of prefrontal cortex, it was shown that serotonin modulates spatial working memory performance via 5-HT1A and 5-HT2A receptors (Cano-Colino et al., 2014). Performance followed an inverted-U relationship, that is, both increases and decreases in serotonin concentrations, [5-HT], led to random choice errors. In their model, 5-HT suppressed pyramidal cell activity via the 5-HT1A receptor by increasing a K^+^ and excited pyramidal cells via 5-HT2A receptors by increasing the Ca^2+^-dependent K^+^ current, which increased intracellular Ca^2+^. The effects of 5-HT on GABAergic interneurons were modeled by inhibiting passive leak currents via 5-HT2A receptors. Another modeling group constructed an efficient spiking neural network model of the dorsal raphe nucleus, which included both serotonergic and non-serotonergic neurons (Wong-Lin et al., 2012). They simulated dorsal raphe nucleus recording experiments from a non-human primate performing a simple perceptual decision task for both rewarding and unrewarding trials (Nakamura et al., 2008; Bromberg-Martin et al., 2010). In addition, to observing the different firing patterns that were found in the primate, the model showed theta band oscillations, especially among the non-5-HT inhibitory neurons, during the rewarding outcome of a simulated trial. In summary, these detailed computational models can allow an investigation of the neural dynamics of serotonergic neuromodulation and its effects on specific receptors. Models at this level may be informative on possible treatments for serotonergic related disorders.

## Models of dopamine and serotonin opponency

It has been suggested that the serotonergic and dopaminergic systems primarily activate in opposition, but at times in concert for goal directed actions (Boureau and Dayan, 2011). Opponency between these systems has been proposed behaviorally and in theoretical models (Daw et al., 2002; Tops et al., 2009). In this notion, dopamine triggers invigorated, reward seeking behavior, and serotonin triggers withdrawn and punishment avoiding behavior. Whether the anatomy supports unidirectional (i.e., the raphe inhibiting dopaminergic areas) or bidirectional inhibition (i.e., raphe inhibiting and being inhibited by dopaminergic areas) is an open issue (Boureau and Dayan, 2011). But there is evidence that projections from raphe serotonin cells to DA areas oppose the actions of DA and mediate avoidance of threats (Deakin, 2003). Interestingly, there is evidence in the striatum that under certain conditions dopamine transporters are able to transport significant amounts of 5-HT into DA terminals (Zhou et al., 2005). These studies suggest that the dopamine and serotonergic systems are highly interactive.

Computational models have been used to investigate these interactions between dopamine and serotonin. One model had tonic serotonin tracking the average reward rate and tonic dopamine tracking the average punishment rate, and that phasic serotonin responses carry a prediction error signal for punishment (Daw et al., 2002). However, it has been difficult to find empirical evidence supporting these roles for tonic and phasic neuromodulation. Modeling has shown that direct opponency between these systems is unnecessary for behavioral opponency (Asher et al., 2010; Zaldivar et al., 2010). In many cases, an environmental tradeoff between expected rewards and costs can lead to opposition between active reward-seeking and withdrawn behavior. Indeed, by having different neuromodulatory systems handle different sensory events, this type of opponency emerged in the present model.

**Figure 6:**
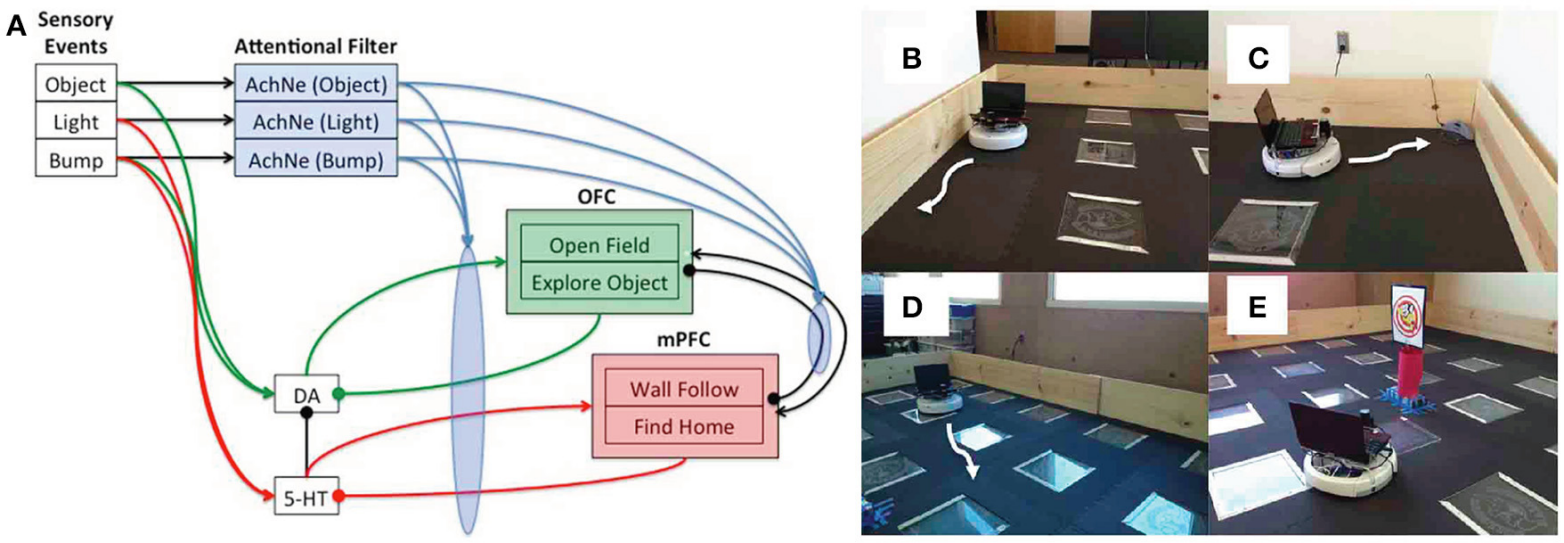
Embodied model of neuromodulation in an open-field test experiment. Experiments were run on an iRobot Create, equipped with a laser range finder and a netbook that contained the neural model and controlled the robot's behavior. **(A)** Neural model architecture. Sensory events were handled by three binary neurons. These neurons projected to the attentional filter neurons (AchNE) and the dopaminergic and serotonergic neurons (DA and 5-HT). The DA and 5-HT neurons projected to the OFC and mPFC neurons. The most active OFC or mPFC neuron dictated the robot's behavioral state. The AChNE neurons had a modulatory effect on the projection from the DA and 5-HT to OFC and mPFC (see blue ellipse and arrows). OFC and mPFC projected to 5-HT and DA neurons with inhibitory connections. Excitatory and inhibitory connections within and between OFC and mPFC neurons were all-to-all. **(B)** Wall following behavior. **(C)** Find home behavior. Finding home consisted of locating the robot's docking station. **(D)** Open-field behavior. The robot moved toward open spaces in the environment based on laser range finder readings. **(E)** Explore object. The robot approached narrow objects based on laser range finder readings. Reproduced from Krichmar (2013) with permission.

A neurorobot model explored the idea of dopaminergic and serotonergic opponency by having the serotonergic system directly inhibit the dopaminergic system (Krichmar, 2013). In this study, he behavior of an autonomous robot in an open-field test paradigm was controlled using a neural network algorithm (see Figure 6). The open-field test is often used in animal models of anxiety (Heisler et al., 1998; Lacroix et al., 2000; Lipkind et al., 2004; Fonio et al., 2009). Similar to mice in the open field test, the robot demonstrated withdrawn, anxious behavior, such as wall following and finding its nest (i.e., the robot's charging station) when serotonin levels were high, and risky, reward seeking behavior, such as moving to the center of the arena or investigating a novel object when dopamine levels were high. Furthermore, the algorithm tested the idea that top-down signals from the frontal cortex to neuromodulatory areas are critical for an organism to cope with both stressful and novel events. As described above, it has been suggested that the mPFC inhibited the serotonergic raphe nucleus after handling a stressful event (Jasinska et al., 2012). This feedback loop prevented the raphe from being overly active after the stressor had been handled. Indeed, when the model's mPFC was lesioned, the robot withdrew to the outer wall or its charging station in response to a stressor such as a bright light or collision. The model further suggested that projections from the OFC to the dopaminergic VTA have a similar function when responding to a positive value event. When the simulated OFC was lesioned, the robot obsessively explored the center of the room and objects in the room. By using a neurorobot experiment that mimics an animal model of anxiety and depression, we can readily observe the behavior in a controlled environment, while also being able to make manipulations that would be difficult in the real animal.

In addition to the studies of serotonin and dopamine in the frontal cortex, interactions between the dopaminergic and serotonergic systems have been observed in the basal ganglia, which may have implications for Parkinson's disease treatments (Bédard et al., 2011). Moustafa and colleagues constructed a neural network model of the basal ganglia, including nuclei such as striatum, subthalamic nucleus and globus pallidum, which were controlled by dopamine and serotonin neuromodulation (Balasubramani et al., 2015). They predict that the modulatory effects of 5HT on dopamine D2 receptors on medium spiny neurons relate to risk sensitivity and reward-punishment learning in the basal ganglia. This may explain risky decision making impairments observed in Parkinson's patients. Moreover, the model suggests that optimizing 5HT levels along with DA medications may improve Parkinsonian deficits in reward-punishment learning.

## Noradrenergic system

With the exception of the basal ganglia, noradrenergic neurons, which originate in the locus coeruleus (LC), project to nearly every cortical and subcortical region (Berridge and Waterhouse, 2003). The LC receives inputs from brainstem structures, but is also highly regulated by the prefrontal cortex, highlighting its role in integrating low-level autonomic and cognitive information and broadcasting this signal throughout the brain. Traditionally the noradrenergic system was thought to mediate arousal levels through slow changes in tonic levels of activation. Phasic activation of the LC, however, characterized by short bursts of activity, has taken on an important role in behavioral adaptation and task performance (Aston-Jones et al., 1994; Aston-Jones and Cohen, 2005).

Phasic activation of the LC typically occurs in response to salient or novel inputs (Sara et al., 1995; Vankov et al., 1995) as well as task-relevant conditioned stimuli. If a reward is not associated with the novel stimulus, the response will eventually attenuate, which is likely important for transitions between phasic and tonic states. Interestingly, the ability of the LC to fire phasic bursts depends on the LC's tonic mode of activation. When tonic activity is either too low or too high, phasic bursts are not present (Aston-Jones and Cohen, 2005). Task performance is optimal when LC neurons can be phasically activated and declines with increasing or decreasing tonic activity. Therefore, an inverted-U relationship between tonic LC activity and task performance exists that resembles the Yerkes-Dodson relationship between arousal levels and task performance. This inverted-U nature of noradrenergic function in terms of signal detection and task performance has also been shown in working memory in the prefrontal cortex (Vijayraghavan et al., 2007; Wang et al., 2007; Avery et al., 2013). That is, too little or too much noradrenaline will likely impair working memory. This, in turn, could lead to attention disorders, stress-related disorders, and obsessive-compulsive disorders.

**Figure 7:**
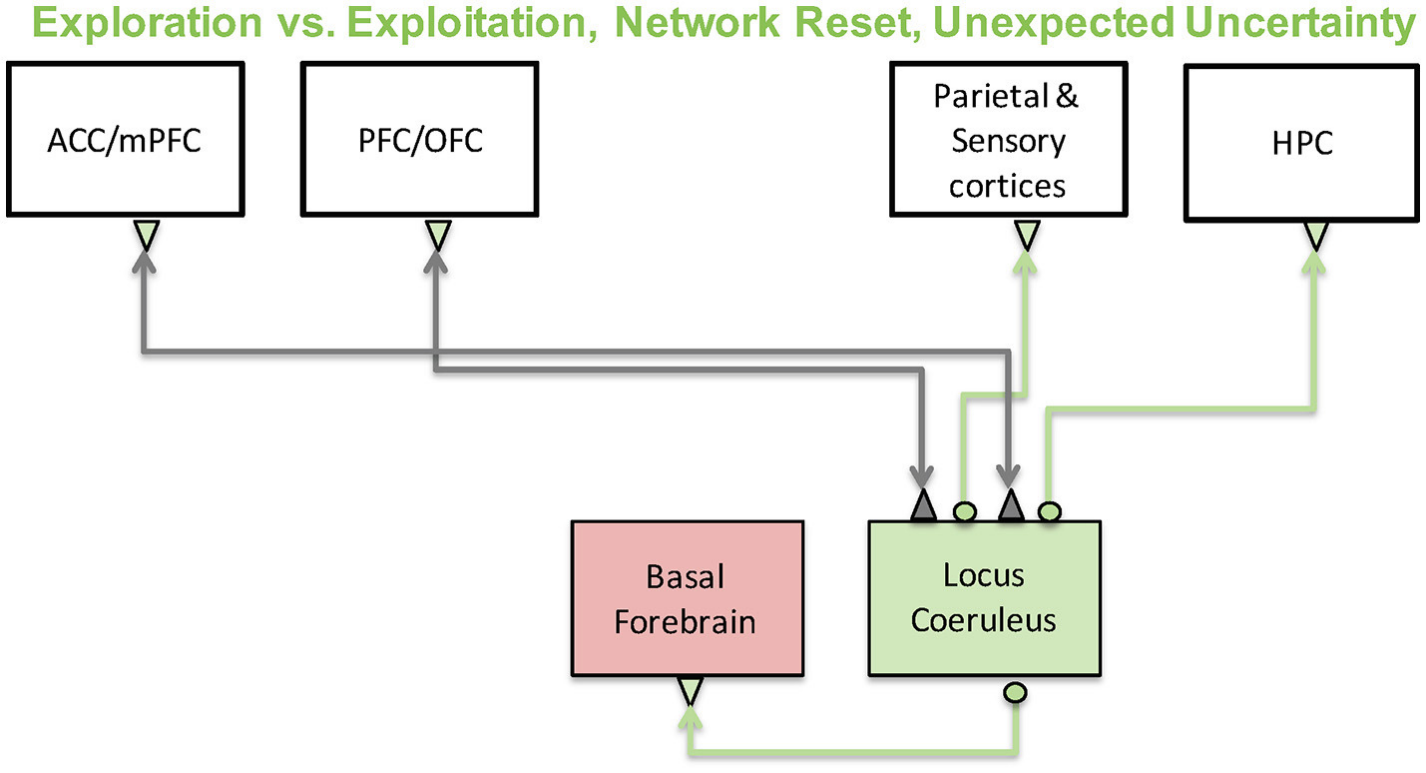
The noradrenergic system and its functions. The noradrenergic system, which originates in the locus coeruleus, has been implicated in exploration-exploitation trade-off computations and large-scale reorganization of networks in the brain in response to surprise. Locus coeruleus activity is regulated by prefrontal and cingulate cortices and sends its projections throughout the cortex as well as to other neuromodulatory regions such as the basal forebrain.

In the past decade or so, two important theories of noradrenergic function have been developed: (1) The “adaptive gain theory” suggests that the noradrenergic system mediates the switch between exploration and exploitation behaviors (Aston-Jones and Cohen, 2005). (2) The “network reset” theory, on the other hand, suggests that the noradrenergic system is critical for functional reorganization of cortical activity when environmental contingencies change to allow for behavioral adaptation (Bouret and Sara, 2005). A schematic depicting the brain regions involved in these computations is shown in Figure 7. We will discuss each of these below as well as recent studies in humans and rodents that have demonstrated an important connection between the noradrenergic system and pupillary responses and how these might be related to cortical states and internal model updating in the brain. Finally, we will discuss a neural network model we recently developed that investigates how varying levels of dopamine and noradrenaline influence working memory and behavior.

## Exploration-exploitation tradeoff

Reinforcement learning theory suggests that at each moment we should act in a way that maximizes reward. The problem with this is that sometimes the algorithm can get stuck in local minimums. The agent may become restricted to a subset of states within the entire space without knowing more rewarding states are possible. In this case, it is advantageous to make locally “non-optimal” actions in order to determine if there are surrounding states that will yield larger rewards. This idea is known as “exploration-exploitation” tradeoff. It has been hypothesized that the noradrenergic system is vital in resolving this computation.

In particular, Aston-Jones and Cohen (Aston-Jones and Cohen, 2005) suggest that exploration and exploitation modes are mediated by tonic and phasic LC activity, respectively. High phasic and low tonic activity is indicative of an exploitive phase in which an animal is task engaged. High tonic modes, however, put the animal into a highly distractible state, allowing them to explore the state space. They propose that the anterior cingulate and orbitofrontal cortices mediate transitions between tonic and phasic LC activity. It is thought that the anterior cingulate plays a role in evaluating cost and conflict, and that the orbitofrontral cortex plays a role in evaluating reward. However, both these regions are implicated in the representation of goal directed behaviors, uncertainty, and outcome expectancies (Schoenbaum et al., 2009; Stern et al., 2010; Gremel and Costa, 2013). More recent work looking at pupillary responses (discussed below) may allow further avenues to test and reshape this theory.

## Network reset, cortical states, and belief updates

The noradrenergic system responds strongly to unexpected changes in the environment as well as task-relevant stimuli, which signal a change in behavior. This has led researchers to hypothesize that phasic activation of the LC is important for a “network reset” that induces a large-scale reconfiguration of neuronal activity across the brain to allow for changes in behavior and cognition (Bouret and Sara, 2005). This has been linked, for example, to the switching between the dorsal attention network, which directs attention to expected stimuli, and the ventral attention network, which attends to novel stimuli (Corbetta et al., 2008). It has also been shown that stress, which directly involves the noradrenergic system, can similarly induce a large-scale reconfiguration of functional activity in the brain and that the reconfiguration is dampened when subjects are given a drug to block adrenergic receptors (Hermans et al., 2011).

The function of the LC in network resetting suggests that it may play a role in internal model updating, which is a well understood computation in a Bayesian framework. Interestingly, pupillary responses, which are strongly correlated to LC activity, are indicative of internal model updating based on Bayesian modeling of human responses. In particular, pupil diameter, in *human experiments*, correlates with learning rates and Bayesian belief updating in a task incorporating predictive inference and uncertainty (Preuschoff et al., 2011; Nassar et al., 2012; Lavín et al., 2014). When a change occurred in the inference task (unexpected uncertainty), pupil diameter increased and correlated with learning rates in their model. This suggests that this new information opened a “gate” to allow new sensory information to affect currently stored priors. More formally, this implies that locus coeruleus may affect the learning rate in Bayesian models as given by the following equation:

(2) $$\begin{array}{l} P_{t+1}=P_{t}+\alpha \cdot \delta \end{array}$$

where P is the prior probability at time t, α is the learning rate and δ is the prediction error as described by reinforcement learning. When the environment is unstable, α will increase to allow for learning and reduce uncertainty. As stability increases, α will decrease so that priors are not updated. The circuit-level mechanism behind this is unknown, however, recent work in the mouse suggests that activation of somatostatin or vasoactive intestinal peptide (VIP) inhibitory interneurons, which disinhibit the cortical or limbic circuit, could gate learning (Letzkus et al., 2015). The noradrenergic and cholinergic systems strongly activate these interneurons, further solidifying their role in uncertainty-related computations. Taken together, these results suggest that the LC may disinhibit circuits to facilitate learning and, simultaneously, improve signal to noise ratios and to allow information to flow smoothly from one region to another when environmental uncertainty is high. Given the LC's link with pupillary responses, it is important to point out that abnormalities in pupillary responses have been associated with a host of disorders including negative symptoms and attentional allocation in schizophrenia (Granholm and Verney, 2004; Granholm et al., 2014), social reward in autism (Sepeta et al., 2012), and reward computations in Parkinson's disease (Manohar and Husain, 2015; Muhammed et al., 2016). Therefore, the LC and pupillary responses may provide a link between investigations of brain disorders and theoretical models of brain function.

Internal model updating may be realized in the brain through cortical state changes, which are also strongly linked to pupillary responses. Cortical states are often associated with oscillatory behavior. For example, low frequency synchronous oscillations are seen in resting states, and asynchronous patterns of activity are seen in active states. Cortical membrane potential recordings show that the transitions between these states occur on the order of seconds and are precisely correlated with pupil fluctuations (Reimer et al., 2014). Moreover, there is an inverted-U relationship between neuronal responses in cortex to sensory cues and behavior that corresponds with pupil diameter (McGinley et al., 2015). When pupil diameter is small, low frequency oscillations exist in the network and there is a high degree of variability in neuronal responses and animal behavior. As the pupil diameter increases, task performance increases concomitantly with sensory-evoked responses while neuronal variability and slow oscillations decrease. Beyond the peak, pupil diameter continues to increase and task performance decreases as gamma oscillations begin to emerge in neurons. Amazingly, much of the variability seen in the membrane potential is directly correlated with pupil fluctuations. These results suggest that sensory information is largely dampened by the brain, however, there is an optimal “window” in which internal “noise” is silenced and sensory events can strongly and reliably drive cortical responses.

The above studies suggest that optimal sensory processing occurs when noradrenergic (NA) levels are neither too high nor too low. This “inverted-U” performance trend is also seen in the prefrontal cortex when primates perform working memory tasks (Vijayraghavan et al., 2007; Wang et al., 2007; Avery et al., 2013). This coincides well with the notion that attention disorders result from the prefrontal cortex being in a “non-optimal” working memory state. Drugs that treat attention disorders, such as guanfacine, which acts on adrenergic α2A receptors, are thought to push the system into an optimal working memory state. We developed a network model of working memory that incorporated this inverted-U feature for dopamine (DA) and noradrenaline (NA) neuromodulation (Avery et al., 2013). The model was of a cortical column with spiking neurons, synaptic conductances, and simulated D1, α2A, and α1 receptors. We simulated the oculomotor delay response task, in which a subject must remember the location of a brief visual cue during a delay period, and then saccade to that location. We explored how changing dopamine and noradrenaline concentrations simultaneously impacts performance and found that working memory is impaired in non-optimal zones, but for different reasons. When NA levels were high and DA levels were low, working memory impairments resulted from excess noise, however, when NA was low and DA was high, impairments resulted from an overall reduction in prefrontal activity. An overall reduction in prefrontal activity is thought to happen during high stress situations and is evolutionarily beneficial in fight or flight situations when “instictual” behaviors need to come online (Arnsten, 2009). If left unchecked, however, stress can ultimately lead to depressive symptoms (Gold et al., 2015). Non-optimal levels in NA may, therefore, play a role in depression and should further be investigated along with the more classic neuromodulators such as dopamine and serotonin. This study highlights the important point that neuromodulatory systems are interconnected and manipulating one system may be useful experimentally, but might not be valid in a real-world setting.

The model described above suggests that optimality in terms of prefrontal processing exists in a higher dimensional space and understanding how multiple neuromodulators interact in different modes (i.e., tonic vs. phasic) could help to expand upon our understanding of attention disorders and cognitive symptoms found in other diseases. Given that frontal regions shape sensory responses, these studies also suggest that different “non-optimal” zones of neuromodulatory activity, which may be associated with unique brain disorders, could manifest as unique changes in sensory processing. In the future it will be interesting to explore how sensory processing and working memory, which are simultaneously shaped by multiple neuromodulatory systems, interact in both healthy and diseased states.

## Cholinergic system

**Figure 8:**
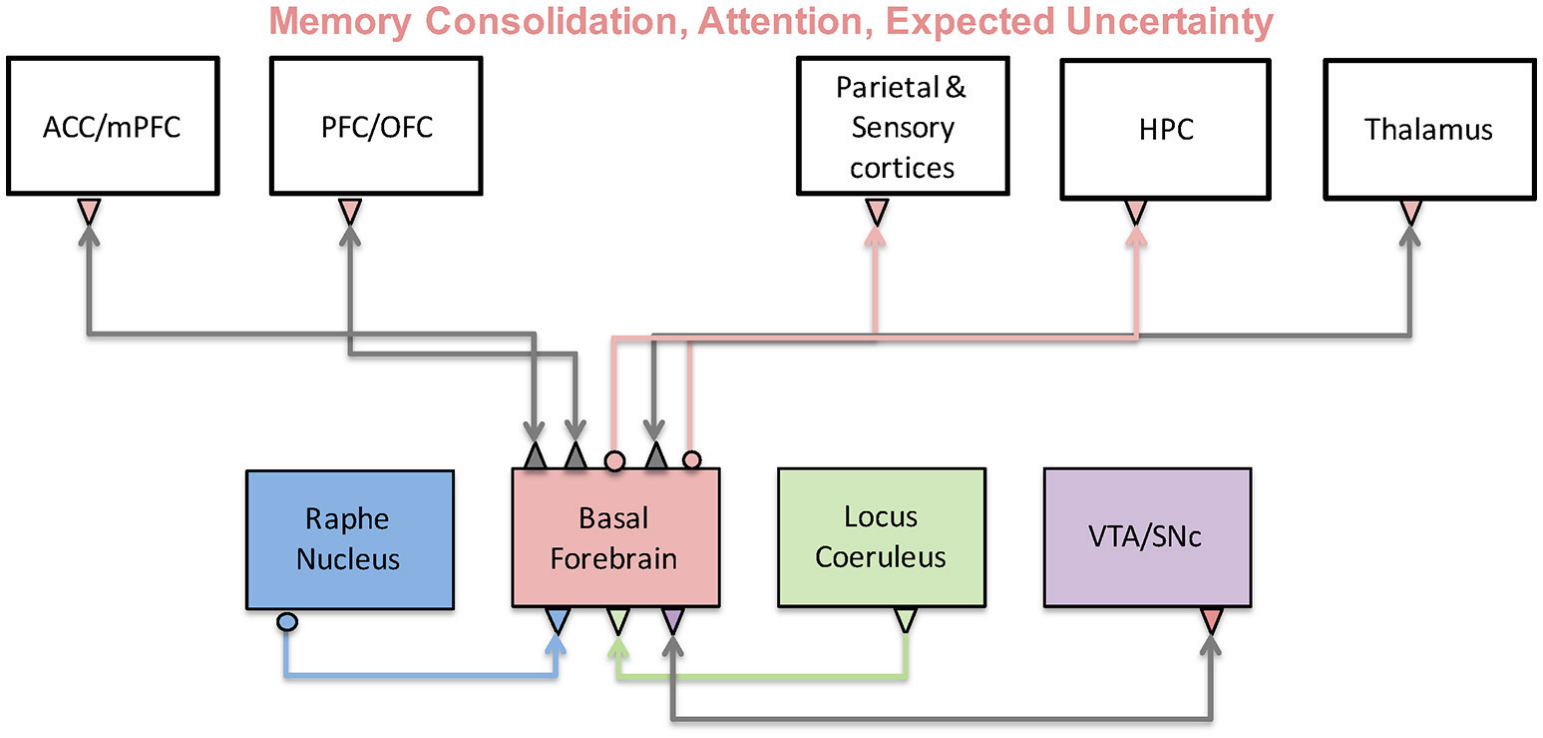
The cholinergic system and its functions. The cholinergic system originates in the basal forebrain and sends projections to many cortical and subcortical regions. As a result, it has been implicated in a variety of functions including memory, attention, and uncertainty computations. Activity of the basal forebrain is thought to be regulated by prefrontal cortices, as well as other neuromodulatory brain regions.

The cholinergic system originates in the basal forebrain and affects essentially every system in the brain including sensory, prefrontal and limbic systems. Research on sleep-wake cycles suggests that a main function of acetylcholine (ACh) plays a major role in memory consolidation (Hasselmo, 1999; Hasselmo and McGaughy, 2004). Hasselmo and colleagues suggested that when ACh levels are low, recurrent connections are stronger and memories are retrieved. But, when ACh levels are high sensory inputs are enhanced, recurrent inputs are reduced, and memory is encoded. Figure 8 shows a schematic of the brain regions and neuromodulators thought to be involved in these memory and sensory functions with the basal forebrain at its center. In particular, it was shown that during slow wave sleep, reduced ACh levels in the hippocampus lead to an increase in recurrent activity relative to cortical inputs, facilitating memory consolidation. While subjects were awake or in REM sleep, however, ACh levels are elevated, leading to an enhancement of cortical input to the hippocampus and stimulating memory encoding. In the following sections, we will mostly discuss conceptual and computational models focused on cholinergic effects on cortical processing. For a recent review discussing modeling cholinergic effects on hippocampus, see Newman et al. (2012).

Attention is strongly modulated by acetylcholine through its projections to sensory cortices (Sarter et al., 2001, 2005). Interestingly, research suggests that the same underlying principle seen in the hippocampus may also hold in sensory cortices. In particular, it is suggested that cortical acetylcholine enhances sensory input relative to recurrent inputs and feedback, leading to an overall improvement in the signal to noise ratio. Cholinergic inputs to visual cortex, for example, have been found to enhance the gain of sensory inputs by stimulating nicotinic receptors located presynaptically on thalamocortical inputs to layer 4 (Disney et al., 2007). Muscarinic receptors have been shown desynchronize population responses and reduce cortical noise by activating somatostatin neurons, which primarily target apical dendrites (Goard and Dan, 2009; Chen N. et al., 2015). Interestingly, muscarinic receptor stimulation has also been shown to enhance attentional signals in the macaque (Herrero et al., 2008), suggesting that the general role of “increasing sensory drive” in the cortex may need to be adapted.

Cholinergic projections to the prefrontal and parietal cortices also seem to play an important role in attention. Cholinergic inputs to these areas play an important role in cue detection (Parikh and Sarter, 2008; Howe et al., 2010) especially when increased attentional effort is required (Bucci et al., 1998; Dalley et al., 2001). Interestingly, prefrontal projections to the basal forebrain can regulate acetylcholine levels in the parietal cortex (Nelson et al., 2005) and may therefore affect the relative salience of targets and distractors (Broussard et al., 2009). A recent study has also implicated decreased nicotinic stimulation to a reduction in frontal lobe activity, termed hypofrontality (Koukouli et al., 2017), which is often associated with the pathophysiology of schizophrenia. This study showed that introducing a single nucleotide polymorphism (SNP) into nicotinic receptors leads to hypofrontality in mice. Moreover, they showed that hypofrontality is alleviated by nicotine administration. This is one of the first studies that establishes a mechanistic link between schizophrenia and nicotine addiction and suggests an important role for the cholinergic system in the pathophysiology and treatment of schizophrenia.

In contrast to cholinergic projections from the substantia innominata to the prefrontal and parietal cortices, which increase attention to salient objects, cholinergic projections from the medial septum to the cingulate and hippocampus are important for decreasing attention to irrelevant stimuli (Chiba et al., 1995; Baxter and Chiba, 1999). In the Baxter and Chiba study, rats with lesioned cholinergic projections to the hippocampus disrupted the animal's ability to decrement attention away from a conditioned stimulus. This pathway for decrementing attention is far less studied than the cholinergic pathway to the cortex and the mechanism behind this is not well understood. It is possible that the decrementing of attention depends on the hippocampus' ability to encode novel information (Hasselmo and Stern, 2006). If attention to a conditioned stimulus should be decremented due to lack of reward, it requires the hippocampus to encode the fact that a reward wasn't present. It is interesting to note that working memory requires interaction between prefrontal cortex and hippocampus, perhaps especially of novel information, suggesting that the incremental and decremental pathways work together to orient behavior in order to learn the value of information in the environment. The importance of ignoring irrelevant information and focusing attention on relevant information is observed in learning disorders such as attention deficit hyperactivity disorder, mild cognitive impairment that lead to dementia, and schizophrenia (for review, see Lubow and Weiner, 2010).

## Cholinergic and noradrenergic computations of uncertainty

The ability to enhance sensory information, decrease recurrent activity, and regulate learning and memory suggests that acetylcholine may have a unique role in uncertainty-mediated inference computations in the brain. A Bayesian statistical theory developed by Yu and Dayan (2002, 2005), indeed, proposes that acetylcholine and noradrenaline levels encode expected and unexpected uncertainty, respectively. These systems, in turn, modulate perceptual inference by balancing sensory and prior information and influencing learning. In a Bayesian statistical framework, the posterior distribution (i.e., perception) is determined by likelihood and prior distributions, which can be thought of, in the context of the Yu and Dayan model, as sensory inputs and top-down expectations, respectively:

(3) $$\begin{array}{l} p\left(h|d\right)=\;\frac{1}{Z}p\left(d|h\right)p\left(h\right) \end{array}$$

Where *p(h|d)* is the posterior distribution of hypothesis given the data, *p(d|h)* is the likelihood function (sensory inputs), *p(h)* is the prior, and *Z* is a normalizing factor. Uncertainty is critical in this model as it determines the relative weight we should assign to priors vs. sensory inputs when making inferences. When prior uncertainty is high, optimal inference entails that sensory inputs should be preferentially weighted and learning should be enhanced so that priors may be updated (also, see discussion in Noradrenergic System section). The same principle also holds when weighting information from different modalities, such as visual and haptic information (Körding and Wolpert, 2006).

The posterior distribution is traditionally solved through exact inference or naïve inference, however, each has its own disadvantages computationally (Yu and Dayan, 2002, 2005). Exact inference requires representing and computing over all possible contexts, making it unlikely to be implemented in neuronal circuits given our current understanding how information is represented in the brain, which is thought to be distributed and inexact (Loftus, 1996; Wixted et al., 2014). Naïve inference does not store prior information over time, making it cheaper computationally than exact inference. Naïve inference, however, leads to poor performance when prior uncertainty is low. The Yu and Dayan model takes a more balanced approach by computing a single state and attaching an uncertainty estimate to this state, which they attribute to the cholinergic signal in the brain. This overcomes the computational disadvantages of the exact inference model and outperforms the naïve model by allowing for use of prior information when uncertainty is low.

The Yu and Dayan model also hypothesizes that phasic bursts of LC activity encode unexpected uncertainty, which can be thought of as a large change in the environment that evokes a “surprise” response. This is consistent with the network-reset theory discussed above in the section on the noradrenergic system. Unexpected uncertainty acts to inform the model that a significant change has happened and priors need to be updated. Inabiltiy to recognize these changes, which can be demonstrated with noradrenergic antagonists, leads to impairments in behavioral flexibility (Caetano et al., 2013). This model assesses reliability in a broader context than the cholinergic encoding of expected uncertainty, which assigns reliability values to individual cues.

**Figure 9:**
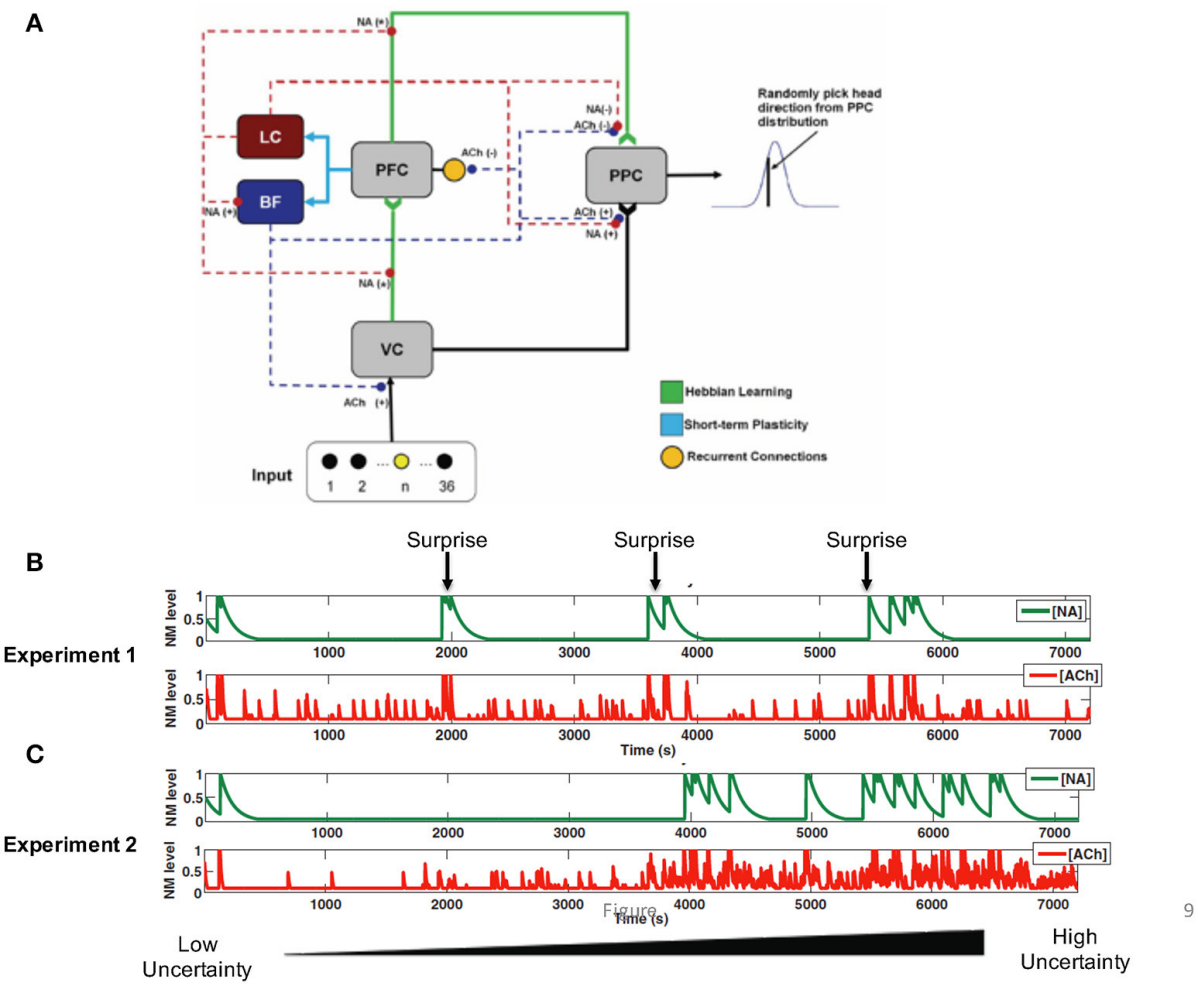
Neural network model incorporating noradrenergic and cholinergic systems that adapt to uncertainty. **(A)** The visual input group drives activity in the VC (visual cortex). VC and PFC (prefrontal cortex) provide input to the PPC (posterior parietal cortex). The noradrenergic system, LC (locus coeruleus), enhances the depression of weights (“forgetting”) between VC and PFC, and PFC and PPC [indicated by NA(−)]. The noradrenergic system increases the gain in the BF (basal forebrain) and the input to the PPC from VC [shown by NA(+)] and suppresses input to the PPC from the PFC [shown by the NA(−)]. The cholinergic system, BF, enhances input to VC and PPC [indicated by the ACh(+)] and suppresses recurrent activity in the PFC and input to the PPC from the PFC [indicated by the ACh(−)]. **(B)** In Experiment 1, the uncertainty level was constant and a surprising stimulus was occasionally presented. NA levels rapidly increased in response to the unexpected stimulus (green), whereas ACh levels rose more gradually. **(C)** In Experiment 2, surprise was kept constant, but expected uncertainty gradually increased. The figure shows that ACh levels increase as expected uncertainty increases (red). Reproduced from Avery et al. (2012) with permission.

In order to understand how Bayesian computations of expected and unexpected uncertainty are realized in the brain, we developed a neural network model (Avery et al., 2012) that incorporated cholinergic and noradrenergic modulation (Figure 9). In particular, we were interested in identifying a mechanism that supports the generation of the noradrenergic surprise response from afferent inputs to the LC and expected uncertainty response through afferent inputs to the BF. Moreover, we hoped to gain insight into how noradrenaline and acetylcholine influence downstream targets to perform Bayesian computations (Avery et al., 2012).

We found that the response of locus coeruleus neurons to novel stimuli and BF neurons to expected uncertainty could be realized in the brain through short-term synaptic depression (Figures 9B,C, blue connections). Short-term plasticity was incorporated into prefrontal projections to the LC and BF. The LC neurons in turn enhanced feedforward input and updated priors by modulating the learning rate of plastic afferent and efferent prefrontal projections. LC neurons also increased the gain of BF neurons as has been shown experimentally (Zaborszky and Duque, 2003). BF neurons, on the other hand, balanced the weight of sensory and prefrontal inputs on decision neurons such that high BF responses favored sensory information. This computational model is unique from many other models of neuromodulation in that it attempts to model both the downstream effects of neuromodulatory input as well as the afferent projections that shape the responses of neurons within neuromodulatory brain regions.

The Bayesian model discussed above suggests that acetylholine computes expected uncertainty in the brain and therefore plays a central role in balancing sensory and prior information. Although we know a great deal about the effects of acetylcholine at the cellular and synaptic levels, this balance of information is likely realized in cortical circuits composed of many neurons of different types in multple brain regions. Deco and Thiele offer insight into this by developing a spiking neural network model that proposes several important mechanisms that mediate the muscarinic enhancement of top-down attention (Deco and Thiele, 2011). Their model incorporated key cellular and synaptic changes resulting from cholinergic modulation including reduction in firing rate adaptation, enhanced thalamocortical input, reduction in lateral connectivity strength, and an increase in inhibitory drive. They show that muscarinic enhancement of attention is mediated by suppression of intracortical connections and an increase in inhibitory drive. Again, this highlights the importance of acetylcholine in suppressing a very specific set of connections (intracortical) and potentially enhancing a broader class of behaviorally relevant inputs, which may include emotional, cognitive or memory.

More recently, we developed a model (Avery et al., 2014) that took a slightly different approach from Deco and Thiele and suggested that local and global activation of the cholinergic system might account for attentional and sensory enhancement, respectively. In this model, stimulation of the basal forebrain has a global effect on the brain and enhances sensory input by disinhibiting the sensory thalamus via inhibitory projections from the basal forebrain to the thalamic reticular nucleus. The model dissociates this enhancement of sensory input from the cholinergic enhancement of top-down input, which suggests that sensory enhancement is mediated by a local release of acetylcholine and activation of muscarinic receptors on inhibitory neurons in the visual cortex. Similar to the Deco and Thiele model, this model stresses the importance of muscarinic receptors on inhibitory neurons. The model demonstrates that activation of muscarinic receptors is primarily involved in reducing noise correlations between neurons, which have been shown to influence information processing capabilities in the cortex. Whether there is local acetylcholine release with attention is still not known. However, (Chen N. et al., 2015) has recently shown the importance of cholinergic activation of somatostatin inhibitory neurons for improving information processing.

The models discussed above aim to understand how sensory and prior knowledge are integrated in the brain. These models, however, do not incorporate learning, which is a key component of cholinergic function and Bayesian models. As discussed earlier, learning to attend toward an object of interest (incrementing attention) and attend away from another stimulus (decrementing attention) is thought to be realized through cholinergic projections to the neocortex and hippocampus/cingulate, respectively. In a neural network model, Oros and colleagues tested the different contributions made by the ACh projections from the substantia innominata/nucleus basalis region (SI/nBM) to the neocortex and the medial septum/vertical limb of the diagonal band (MS/VDB) in incrementing and decrementing attention. The neural simulation was tested in a range of behavioral paradigms that require both attending to a salient stimuli and ignoring an irrelevant stimuli (Oros et al., 2014). The model exhibited behavioral effects such as associative learning, latent inhibition, and persistent behavior. The model suggests that the neuronal projection from the MS/VDB to the hippocampus and cingulate is important for: (1) Decreasing attention to a cue that previously predicted a reward. (2) Preventing perseverative behavior when reward contingencies change (e.g., in extinction or reversal learning tasks). (3) Showing latent inhibition to previously uninteresting cues. Lesioning the MS/VDB disrupted latent inhibition, and drastically increased perseverative behavior. Taken together, the model demonstrated that the ACh decremental pathway originating in the MS/VDB is necessary for appropriate learning and attention under dynamic circumstances and suggests a canonical neural architecture for attention that includes both an incremental and a decremental pathway.

## Conclusions

The present article reviewed experimental evidence, as well as computational and theoretical models of neuromodulation. It is difficult to pinpoint a specific function for each neuromodulator. It has been suggested that dopamine is related to positive value, serotonin to risk aversion, noradrenaline to vigilance, and acetylcholine to attentional effort (Krichmar, 2008). Another theory posits that dopamine is related to reward prediction, while serotonin is related to temporal discounting, and that noradrenaline regulates the exploration/exploitation tradeoff, while acetylcholine controls learning rate (Doya, 2002, 2008). These functions can be mapped to elements of temporal difference learning. However, in neither case are things this simple. The same neuromodulator can have different effects on their target brain areas. For example, dopamine has different functional implications depending on whether it targets D1 or D2 receptors (Durstewitz and Seamans, 2008; Avery and Krichmar, 2015). Acetylcholine increments attention in sensory cortex, but decrements attention in the cingulate and hippocampus (Chiba et al., 1995; Baxter and Chiba, 1999; Oros et al., 2014). Interestingly, all neuromodulators are involved to some degree in attention and novelty detection. This suggests that no matter what the specific function, neuromodulators in all cases signal important events for the organism and shape behavior.

In this review, we highlight studies that focus on the interactions within and between neuromodulatory systems. Still, most of the experiments, computational models and theoretical models described here focused on one or two neuromodulators. There are strong interactions between all of these systems. An exploratory survey of cholinergic, dopaminergic, noradrenergic, and serotonergic receptor expression using the Allen Mouse Brain Atlas showed that the substantia innominata of the basal forebrain, which is a source of cholinergic innervation, and the VTA, which is a source of dopaminergic innervation, displayed high receptor expression of all four neuromodulators (Zaldivar and Krichmar, 2013). Since the nuclei of these neuromodulatory systems are thought to be the source of specific neurotransmitters, the projections from these nuclei to target regions may be inferred by receptor expression and suggest that neuromodulatory systems are highly interactive. It should be noted that many of these nuclei, in which neuromodulatory neurons originate, also have GABA-ergic and glutamatergic neurons (Zaborszky, 2002; Barker et al., 2016). Moreover, there is evidence that multiple neurotransmitters and neuromodulators are co-released at the axon terminals of these neurons (Trudeau, 2004; Sarter et al., 2005; Zhou et al., 2005). We have a limited understanding of how these interactions affect the functionality of the nervous system. Therefore, more computational, theoretical and disease models that focus on these interactions are needed. Theoretical models are important and can help us reduce and simplify these complex interactions in terms of a single overarching computation, such as computing uncertainty.

Computational models of neuromodulation and its effects can contribute to our understanding of a number of neurological diseases and disorders. Dopamine's involvement in schizophrenia has been modeled many times (Braver and Cohen, 1999; Loh et al., 2007; Durstewitz and Seamans, 2008; Rolls et al., 2008; Arnsten et al., 2012; Avery et al., 2012), as well as Parkinson's disease (Moustafa and Gluck, 2011; Moustafa et al., 2013). Serotonin is thought to be involved in anxiety disorders (Millan, 2003; Tops et al., 2009; Jasinska et al., 2012) and depression (Deakin, 2003; Weisstaub et al., 2006; Gold et al., 2015). Models of anhedonia, anxiety, and withdrawal can provide mechanistic underpinnings for these disorders (Wong-Lin et al., 2012; Huys et al., 2013; Krichmar, 2013). The cholinergic and noradrenergic systems play a significant role in allocating attention, and models of these systems may have implications on how imbalances in these neuromodulators can contribute to ADHD (Yu and Dayan, 2005; Cohen et al., 2007; Deco and Thiele, 2011; Avery et al., 2013, 2014). Many of the current drug treatments for these disorders target neuromodulators. Thus, understanding how these drugs can disrupt the fine balance in neural circuits through computational modeling is of the utmost importance.

Detailed computational models will be important for understanding the complexity of neuromodulation including how neuromodulatory responses are generated (e.g., short-term plasticity Avery et al., 2012), the result of influencing multiple targets simultaneously, how neuromodulatory systems interact with each other directly, and how these systems interact in target sites. We hope that computational and theoretical models may work hand in hand with experimental research to drive discovery of the underlying mechanisms a large set of multifaceted and complex disorders.

## Author contributions

All authors listed have made a substantial, direct and intellectual contribution to the work, and approved it for publication.

### Conflict of interest statement

The authors declare that the research was conducted in the absence of any commercial or financial relationships that could be construed as a potential conflict of interest.
